# Exploring the impact of shielding advice on the wellbeing of individuals identified as clinically extremely vulnerable amid the COVID-19 pandemic: a mixed-methods evaluation

**DOI:** 10.1186/s12889-022-14368-2

**Published:** 2022-11-22

**Authors:** Gemma Lasseter, Polly Compston, Charlotte Robin, Helen Lambert, Matthew Hickman, Sarah Denford, Rosy Reynolds, Juan Zhang, Shenghan Cai, Tingting Zhang, Louise E. Smith, G James Rubin, Lucy Yardley, Richard Amlôt, Isabel Oliver

**Affiliations:** 1 NIHR Health Protection Research Unit (HPRU) in Behavioural Science and Evaluation, Bristol Medical School, Population Health Sciences, University of Bristol, Bristol, BS8 2BN UK; 2 Field Epidemiology Service, UK Health Security Agency, Cambridge, UK; 3 Field Epidemiology, Field Service, National Infection Service, UK Health Security Agency, Liverpool, UK; 4 NIHR Health Protection Research Unit in Emerging and Zoonotic Infections, University of Liverpool, Liverpool, UK; 5 NIHR Health Protection Research Unit in Gastrointestinal Infections, University of Liverpool, Liverpool, UK; 6 School of Psychological Science, University of Bristol, Bristol, UK; 7 Population Health Sciences, Bristol Medical School, University of Bristol, Bristol, UK; 8 NIHR Health Protection Research Unit in Emergency Preparedness and Response, King’s College London, London, UK; 9 Department of Psychological Medicine, King’s College London, London, UK; 10 Psychology Department, University of Southampton, Southampton, UK; 11 Behavioural Science and Insights Unit, UK Health Security Agency, Salisbury, UK

**Keywords:** COVID-19, Shielding, Infection control, Health policy, Public health

## Abstract

**Background:**

The national shielding programme was introduced by UK Government at the beginning of the COVID-19 pandemic, with individuals identified as clinically extremely vulnerable (CEV) offered advice and support to stay at home and avoid all non-essential contact. This study aimed to explore the impact and responses of “shielding” on the health and wellbeing of CEV individuals in Southwest England during the first COVID-19 lockdown.

**Methods:**

A two-stage mixed methods study, including a structured survey (7 August—23 October 2020) and semi-structured telephone interviews (26 August—30 September 2020) with a sample of individuals who had been identified as CEV and advised to “shield” by Bristol, North Somerset & South Gloucestershire (BNSSG) Clinical Commissioning Group (CCG).

**Results:**

The survey was completed by 203 people (57% female, 54% > 69 years, 94% White British, 64% retired) in Southwest England identified as CEV by BNSSG CCG. Thirteen survey respondents participated in follow-up interviews (53% female, 40% > 69 years, 100% White British, 61% retired). Receipt of ‘official’ communication from NHS England or General Practitioner (GP) was considered by participants as the legitimate start of shielding. 80% of survey responders felt they received all relevant advice needed to shield, yet interviewees criticised the timing of advice and often sought supplementary information. Shielding behaviours were nuanced, adapted to suit personal circumstances, and waned over time. Few interviewees received community support, although food boxes and informal social support were obtained by some. Worrying about COVID-19 was common for survey responders (90%). Since shielding had begun, physical and mental health reportedly worsened for 35% and 42% of survey responders respectively. 21% of survey responders scored ≥ 10 on the PHQ-9 questionnaire indicating possible depression and 15% scored ≥ 10 on the GAD-7 questionnaire indicating possible anxiety.

**Conclusions:**

This research highlights the difficulties in providing generic messaging that is applicable and appropriate given the diversity of individuals identified as CEV and the importance of sharing tailored and timely advice to inform shielding decisions. Providing messages that reinforce self-determined action and assistance from support services could reduce the negative impact of shielding on mental health and feelings of social isolation.

**Supplementary Information:**

The online version contains supplementary material available at 10.1186/s12889-022-14368-2.

## Background

On 22 March 2020, the Secretary of State for the UK Government announced that individuals in England who, based on understanding at the time faced the highest risk of being hospitalised by COVID-19, should “shield” themselves [1]. Members of this group were initially advised to not leave their homes for 12 weeks and not go out for shopping, travel, or leisure. This marked the start of what came to be known as ‘shielding’ in England, which was later paused on 1 August 2020.

At the start of the pandemic, the UK Government identified the need to develop a patient list of clinically extremely vulnerable (CEV) people so that they could be sent public health advice and offered support to stay at home and avoid all non-essential contact [1]. Those classified as CEV comprised people of all ages with specific health conditions, certain cancers, and organ transplant recipients, plus individuals identified by their General Practitioner or hospital specialist as being at a higher risk from SARS-CoV-2 infection [2, 3]. However, as there was no single mechanism available to support this identification process, a challenging and complex clinical data search was conducted across primary and secondary care settings in England. In total 2.2 million individuals were formally identified as CEV, but various delays were reported in identifying, communicating, and supporting CEV people during this initial period [1]. For those who were able to officially register with the UK Cabinet Office as CEV [4], support with food, medicine, and basic care was offered by central government, local authorities, service providers, charities, rapidly-formed local support groups, neighbours, and relatives.

During the initial 12-week period of shielding over 500,000 people were provided with government-funded food parcels [1]. The shielding advice provided in England suggested a unique combination of behaviours for CEV individuals, which included strictly avoiding contact with anyone displaying coronavirus symptoms, staying at home, not attending any gatherings, not going out for shopping, leisure or travel, and arranging for food and medication deliveries to be left without social contact. Inside the home, shielding people were advised to minimise time spent with others in shared spaces, keep two metres away from others and sleep in a different bed, use a separate bathroom if possible, and avoid using the kitchen when others were present, eating in a separate room. This specific set of guidelines was distinct from the social distancing and quarantine requirements imposed on the UK general population during the same time period [5].

The shielding advice combined various types of non-pharmaceutical interventions (NPIs) that are established components of the public health responses to outbreaks [6–10]. Yet attitudes towards such pandemic-related social interventions are known to vary among subgroups as documented by evidence for a range of positive and negative perceptions [11], and the extent to which they effectively reduce risk depends largely on the willingness and ability of the population to adhere to NPIs [12]. Our understanding of the impact of extended periods of shielding is limited. The closest analogies in the literature relate to quarantine. These studies show stay-at-home and social distancing interventions used during the 2003 SARS and 2009 H1N1 pandemics were associated with detrimental mental health effects, especially in vulnerable populations that may require additional support [13–17]. More recently, COVID-19 related studies found that disruption to normal routines (e.g., academic delays, stopping work) was associated with increased anxiety and psychological distress [18, 19]. However, quarantine tends to be shorter, often involves different groups of people, and has a different purpose (the protection of others, rather than the protection of yourself). Given the unique nature of the official shielding advice distributed by the UK Government in March 2020, the aim of this study was to gain a better understanding from individuals identified as CEV about the effectiveness and acceptability of advice that they received to “shield” during the COVID-19 pandemic, and to explore the reported impact of shielding on their wellbeing.

## Methods

A two-stage mixed methods study, including a structured survey and semi-structured telephone interviews with a sample of individuals who had been identified as needing to “shield” by Bristol, North Somerset & South Gloucestershire (BNSSG) Clinical Commissioning Group (CCG).

### Patient and public involvement

Given the extremely rapid and responsive nature of this research, it was not possible to involve patients or the public in the development of the study and associated materials. However, clinical staff at BNSSG CCG were involved in planning the study and facilitating participant recruitment. Additionally, preliminary results from this study were discussed with members of the BNSSG CCG during analysis and the findings will be shared with participants on publication.

### Structured survey

A random sample of 840 people were contacted by post and invited to take part in the structured survey, stratified by index of multiple deprivation (IMD [20]; 240 in the lowest quintile and 150 in each of the remaining quintiles (600 in total)). Potential survey participants were given the option to respond via post or online.

All surveys were completed between 7^th^ August 2020 and 23^rd^ October 2020. The survey consisted of a 54-item questionnaire, including sections on sociodemographic and household characteristics, knowledge of coronavirus symptoms and public health advice, self-reported barriers and facilitators to advice and a self-assessment of mental health and wellbeing. The Patient Health Questionnaire (PHQ-9) [21] was used to screen for probable depression and the Generalised Anxiety Disorder Scale (GAD-7) [22] was used to screen for probable anxiety, both using a cut-off point of 10 to indicate the possibility of clinical presentation. The PTSD checklist (PCL-5) [23] was used to screen for possible post-traumatic stress disorder (PTSD), using a cut-off point of 4 to suggest a potential clinical presentation (full survey available in Supplement 1).

Data from each survey were initially analysed using summary statistics. Not all respondents answered all questions, therefore percentages given below use the number of respondents to each question as a denominator. Where relevant, variables were consolidated into binary indices and compared using a chi-squared test of independence. Statistical analyses were performed in R and RStudio (V1.1.463) [24].

### Qualitative interviews

Interviews were conducted between 26^th^ August and 30^th^ September 2020. At the time of interviews, all four UK nations had relaxed their lockdown measures. Non-CEV members of the general public living in England could return to work if their workplace was considered COVID safe. Non-essential shops and places of worship reopened, but strict social distancing was encouraged (i.e., staying two metres apart). Groups of six individuals from different households were allowed to meet outside. Anyone with COVID-19 symptoms, and their household contacts, were expected to isolate. Most notably, for the purposes of this study, individuals identified as CEV were advised to remain cautious and to stay at home where possible and, if they did go out, to follow strict social distancing.

All responders to the survey were eligible to participate in a qualitative interview. Forty-five respondents consented to take part in a follow-up interview, but not everyone who consented responded to subsequent communication. This homogeneous sample of CEV individuals consisted of those that completed the survey, [25], shared valid contact information used to identify interview participants, and consented to participate in the interview. In total 45 potential interviewees were initially approached via email and 14 responding individuals were subsequently emailed an information sheet about the study. In total, due to a loss at follow-up, 13 interviews were conducted via telephone (GL). Participants were offered a £20 shopping voucher as reimbursement for their time.

Interviews lasted between 42 to 69 min (median 54). Verbal consent from participants was recorded. A flexible topic guide developed using grounded theory approach was used to aid questioning, allowing participants to discuss emerging ideas [26]. Participants were asked about the shielding advice they had received during the first UK lockdown (23^rd^ March 2020 to 1^st^ August 2020), the acceptability of this advice and their resulting behaviours (Supplement 2).

Interviews were transcribed, anonymised, and thematically analysed using NVivo 12 (QSR International) [27]. A subset of transcripts were coded inductively to establish an initial analysis framework (CR). This framework was then applied (GL) while reading each transcript and listening to the interview audio files to help capture verbal emphases. Coding was performed iteratively within and between transcripts, using a constant comparative method [28]. Following initial thematic analysis, two researchers (GL and PC) independently coded a selection of transcripts. Themes relating to participants’ understanding and adherence to the UK Government’s shielding advice were discussed, plus reported experiences and behaviours during the 12-week lockdown. Data were compared to the initial coding framework, with adaptations discussed, agreed, and made as required. The constant comparison between data and analysis allowed the development of codes, categories, and theories to be tested across transcripts, using a grounded theory approach to identify key themes [29].

Analysis of the interviews was conducted separately from the survey analysis. Data triangulation was used, whereby the data generated from the two methods were systematically reviewed and brought together by identifying common themes.

### Research ethics approval

Ethical approval for this study was obtained on 27^th^ May 2020 from the Heath Research Authority and Health and Care Research Wales (Project ID 284,629, REC ref 20/HRA/2549).

## Results

### Survey and interview participation

Two hundred and three respondents completed the survey (Table 1). Most (110, 54%) respondents were over 69 years old, 75 (37%) were between 45 and 69 years old and 18 (9%) were between 25 and 44 years old. One hundred and ninety-one (94%) respondents identified as White British. One hundred and eighty-four respondents gave their occupation: one hundred and twenty-eight (64%) were retired, 22 (11%) working full time, 16 (8%) working part-time, seven (4%) were currently on leave or furloughed, seven (3%) were unemployed and four (2%) were stay at home parents / housemakers. 70% of survey responders lived with their family, 1% shared their property with non-family members and 29% lived alone. Nearly all survey responders (97%) had access to outside space at home, such as a garden, yard, balcony or terrace.

**Table 1:** Demographic information for survey respondents and interview participants

Characteristic	Survey respondents		Interview participants	
	**n**	**%**	**n**	**%**
Gender (number of responses to question)	203	99.5		
Male	86	42.4	6	46
Female	117	57.6	7	54
Age (number of responses to question)	203	99.5		
25 to 44	18	8.9	3	23
45 to 69	75	36.9	4	31
69 +	110	54.2	6	46
Ethnic group (number of responses to question)	203	99.5		
White-British	191	94.1	13	100
Mixed	4	2.0	0	0
White-other	2	1.0	0	0
Asian	2	1.0	0	0
Black or Black British	1	0.5	0	0
African	1	0.5	0	0
Chinese	1	0.5	0	0
Other	1	0.5	0	0
Current employment status (number of responses to question)	200	98.0		
Retired	128	64.0	8	62
Working full time	22	11.0	2	15
Usually working full time, currently on leave/ furloughed	4	2.0	0	0
Working part-time	16	8.0	2	15
Usually working part-time, currently on leave/ furloughed	3	1.5	0	0
Stay at home parent or homemaker	4	2.0	1	8
Unemployed	7	3.5	0	0
Other	16	8.0	0	0
Household composition (number of responses to question)	200	98.0		
Live with family	141	70.5	8	62
Live alone	57	28.5	5	38
Share with non-family members	2	1.0	0	0
Access to outside space at home (garden, yard, balcony or terrace) (number of responses to question)	202	99.0		
Yes	195	96.5	12	92
No	7	3.5	1	8

A total of 13 survey responders (7 female) took part in the interviews (Table 1). All interviewees were White British, with ages ranging from 25 to over 69 years. Most (8, 62%) of interview participants were retired, four were in either full or part-time work and one was a fulltime parent. One interviewee reported having no access to outside space at their home and five participants lived alone.

### Acceptability of official advice to “shield”

Most respondents to the survey (80%) agreed or tended to agree that they received all information required, and most (76%) thought they tried to initially follow all advice related to shielding. People who thought they would try to follow all advice related to shielding initially were more likely to feel that they received all the information they needed (X^2^ = 7.396, df = 1, *P* = 0.007).

When explored during interview the initial timing of shielding advice was questioned by some participants, especially those who had already begun shielding before they received any official advice (Table 2, Quote 1). Interviewees reported receiving official shielding advice at various times between March and June 2020. Two participants reported never receiving an ‘official letter’ from the government advising them to shield, rather they received confirmation of their CEV status from a variety of sources such as their healthcare provider or local council. These inconsistencies were problematic for some CEV interview participants, as it meant that they did not receive any formal advice until midway through their shielding period, which ran from 23^rd^ March – 1^st^ August 2020 (Table 2, Quote 2).

**Table 2:** Acceptability of official advice to “shield” to interview participants

Quote 1	“I heard on the news in February that this was around, I heard it in January actually, I locked myself in because I knew what was going to happen. So, the actual notification from the NHS was the 23^rd^ March at which time I’d already locked myself in for about four weeks at that time” (Shield 5)
Quote 2	“This was the first official notification we had from anybody about [shielding], and it’s dated 22 June. It’s from [local authority organisation] saying that, ‘You were sent a letter from the government as you have been identified as being extremely vulnerable’. Well, the answer to that was, well, no, we didn’t” (Shield 10)
Quote 3	“We did watch the broadcast every day on the television from the government because my husband found it fascinating. I got a bit lost because it’s so complicated…” (Shield 9)
Quote 4	“I started to watch some of the daily briefings from the government and then stopped because I was becoming a bit… I thought, oh God, I’ll never leave my house ever if I keep watching these! [laughs] So, you know, it became a bit depressing” (Shield 7)
Quote 5	“Watching it on the television sometimes with the announcer like Boris, I’d get confused because they’d say something and then they’d change It” (Shield 9)
Quote 6	“Daily briefings from the government, it almost felt a bit flippant that people like me were told to shield…. I felt a bit as if we were pushed to one side and it’s quite fine that your life is put on hold and you have to stay in your house… let’s get everything else back to normal but forget about the shielders, they can just stay at home out of the way” (Shield 12)

After the initial notification to shield, most interview participants noted that they frequently sought additional information to supplement the shielding advice. The daily Government televised briefings were identified as a key information source for all interview participants at the start of the pandemic, although these updates were increasingly supplemented with information from other sources (e.g., radio, newspapers, internet searches, social media, family members, and friends) due to concerns about the comprehensibility, relevance, and consistency of these daily briefings (Quote 3 to 5). When reflecting on the televised briefings a few interview participants talked about the updates and advice not being relevant to shielding CEV individuals and that this led to feelings of being forgotten or disregarded (Table 2, Quote 6).

### Attitudes and behaviours in response to shielding advice

Two thirds (66%) of the survey responders reported that all people in their household shielded with them by staying at home to avoid contact with others, and further 21% said that other household members tried to shield with them but were not able to. Members of the household were more likely to decide to shield together if all of them were over 70 or CEV (88% compared with 54% if least one person in the household was under 70 and / or not CEV) (X^2^ = 17.16, df = 1, *P* < 0.001). Interview participants reported that the shielding advice and perceived risks of COVID-19 infection were considered when deciding on their shielding behaviours and for some, shielding as a household was felt to be the only realistic approach, otherwise they would have been unable to shield in accordance with the government’s advice due to restricted living space or caring responsibilities (Table 3 – Quote 1).

**Table 3:** Interview participants’ attitudes and behaviours in response to the shielding advice

Quote 1	Primarily “The governments advice, to be honest that whilst it was comprehensible, it wasn’t necessarily easy to follow. Which is basically, if you were shielding and you’re living with somebody else who has not been asked to shield, the advice then went on to say you need to keep two metres distance from them at all times. Ideally sleep in different bedrooms, make different arrangements for your meals, use different toilet facilities etc. etc. And we read this through, and we said well we couldn’t actually do that over an extended period of time. It would be absolutely awful you know we’re a married couple. We have a shared life; we couldn’t be basically isolating from each other within the house for weeks on end… and I said to ((spouse)) just think about it if we’re both shielding together there is no need to do that if we are both taking exactly the same precautions, we’re not introducing risk into the household… so that was the basis on which we decided largely we were going to shield together” (Shield 3)
Quote 2	“I think you kind of had to follow the guidelines really. I just tweaked them in a bit of a way that I guess suited me at the time. I read the bits that I wanted to read, and I believe I kept to the guidelines as sufficiently as I could for me” (Shield 4)
Quote 3	“As lockdown progressed, I found the urge to go out. I mean I’d be ultra-cautious; I wouldn’t go out into crowds; I’d go out at odd hours when nobody was around. I’d wear nitrile gloves and face covering but it was something I had a need to do just to get out from the four walls that sort of it became a bit of a prison I suppose. And I exercised that sort of covert, that sort of outside activity, I felt like I was creeping out put it that way and I did that about once maybe twice a week maximum.” (Shield 1)
Quote 4	“[Local] council were fantastic. After I’d registered as shielding I had a phone call from somebody to say ‘You know we’re here if you need us ring through?’ etc. etc. and that was really good and then they followed up a couple of weeks later saying ‘Is everything okay?’” (Shield 3)
Quote 5	“I found the community in my street really helpful. I didn’t call upon them a lot but it was lovely to know that they were there if I needed them. I found it absolutely vital that we were prioritised for supermarket slots, that would have been very difficult if we hadn’t had that.” (Shield 13)
Quote 6	“We welcomed the fact that we were legitimised to shield so it’s had a positive effect. And also it sounds a strange thing to say in a national emergency but in a strange kind of way we’re actually certainly for the first couple of months quite enjoyed it because not only was the relief okay we can now sort of stay safe but there was also the fact that right now we’ve got chance to get on top of all sorts of projects and things that we’ve been meaning to do for a long time but maybe haven’t found time” (Shield 3)
Quote 7	“I don’t want to shield again, that’s one thing I know, I don’t want to do it. I will definitely moderate my behaviour for going out, but I don’t want to shield. I don’t want anybody to say to me again, you are locked in your home for the foreseeable future” (Shield 12)

None of the interview participants fully adhered to all the shielding recommendations; indeed, a spectrum of shielding adherence behaviours were reported, with advice being adapted to suit personal circumstances (Quote 2) and found to wane over time (Quote 3). Key external factors found to impact on the shielding behaviours of participants were access to outside space, food, medication supplies, and community support. In the latter case, for some interview participants the community support they received during shielding was reassuring and allowed them to “maintain shielding” (Shield 3). However, survey results showed that the types and level of support received by CEV individuals varied. For example, since being told to shield, 63% of survey responders reported receiving help that they had not needed previously. Most survey responders reported asking for additional support, mainly from family (135; 66.2%) and / or friends (48; 23.5%), and for those that received support it was chiefly from family (148; 73.3%) and / or friends (53; 26.2%). 92.1% (35/38) of people who requested organisational support (e.g., NHS, council, government, charity) during shielding received it. When explored during interview, some discussed unsolicited offers of support from their local council to help with medication and food deliveries (Quote 4) and most talked about supplementing this with ad hoc help from family, friends, or neighbours (Quote 5). None of the qualitative interviewee participants felt they could have followed the shielding advice without some external community support, as the recommended combination of NPIs was considered highly restrictive.

Despite some of the difficulties reported when following the shielding advice, 71% of survey responders felt that they were likely or highly likely to follow similar shielding advice for another three months if needed. A few interview participants even described the label of “shielding” as socially advantageous, as it legitimised their decision to self-isolate (Quote 6). However, over half of survey responders (56%) thought that it would be hard or very hard to follow such additional restrictions, indeed some interview participants had strong reservations about needing to shield again in the future (Quote 7).

### Impact of “shielding” on health and wellbeing

#### Accessing healthcare

Half of survey responders (51%) had successfully accessed healthcare either virtually or in person since being advised to shield, however an additional 13% experienced some problems. The remaining 36% had not tried to access healthcare. Interviewees also recounted mixed experiences, with some reporting smooth interactions with their healthcare providers, others experiencing initial breakdowns in communication with their GP practice or secondary care specialists that were quickly resolved and a minority having no healthcare interactions during shielding.

Survey responders were presented with a list of possible symptoms that could be attributed to COVID-19. Approximately half of the survey respondents (47%) reported at least one of these symptoms since being advised to shield. The most common signs reported were non-specific: “feeling tired or having low energy” (38%) and “aches and pains” (19%). Approximately a quarter (27%) of people with symptoms sought professional help either on the phone (24%) and /or in person (11%). Assuming that phone calls preceded a visit to a healthcare facility, only two people did not call a healthcare provider prior to visiting a healthcare facility. 42% of survey respondents that reported symptoms modified their behaviour to decrease contact with other people inside and / or outside their household.

#### Impact on physical health

Most survey respondents (81%) did not think, or were sure, they had not had coronavirus, 12% were unsure if they had had it, and 7% thought they had probably or definitely had coronavirus.

Most survey respondents answered that they had a health problem that limited their activities prior to being asked to shield (71%). Despite this, from all survey respondents 67% did not need regular help, 68% did not have health problems that required them to stay at home and 72% did not need to use a stick, walker, or wheelchair to move about (72%). Respondents aged over 69 were more likely to answer yes to at least one of these questions than younger respondents (X^2^ = 4.607, df = 1, P = 0.032). The majority of those surveyed thought that shielding had no impact on their physical health, although more surveyed people thought that shielding was making their physical health worse (11% strongly agreed and 26% tended to agree) than thought shielding was making their physical health better (5% strongly agreed and 6% tended to agree). Furthermore, some interviewees felt that shielding had negatively impacted on their levels of daily exercise (Table 4—Quote 1).

**Table 4:** Impact of “shielding” on health and wellbeing of interview participants

Quote 1	“It hasn’t done much for my physical health… and I suppose towards the middle I was, sort of losing a bit of motivation… I don’t think I was getting the same kind of exercise I was getting before, so that’s not great” (Shield 7)
Quote 2	“I think when you’re stuck in the house and someone has told you that you cannot leave your home, you feel a bit trapped. I am really lucky, I have quite a big house, I’ve got a lovely garden and we had nice weather so I could go outside but it’s just not the same as going out and interacting with other people and just getting a bit of fresh air… When you are told for a really prolonged period that you’ve got to stay at home, it does make you feel like a caged animal because you’re trapped in the house. You can't speak to anybody, you can't go out or do anything.” (Shield 13)
Quote 3	“I couldn’t practically shield from [our children] but they still needed to have regular exercise and be outside, so that caused some anxiety.” (Shield 13)
Quote 4	“I’d probably have another course of medication to treat the mental illness I could have suffered and I’m not exaggerating that one I don’t think. No, I would have gone you know it was bordering on the crazy I think just being locked up for three months in isolation it wasn’t going to be for me.” (Shield 1)
Quote 5	“I seemed to get more emotional. I tend to worry a lot and then when you watch the news and you see people in – and I think, why am I worrying, because they’ve got a far worse deal than me? So, I’ve got to sort my head out.” (Shield 9)
Quote 6	…as someone shielding, some of the advice – especially as things started to relax a little for the general population – it became a bit depressing actually.” (Shield 8)
Quote 7	“The government’s bumbled along and hasn’t really got a grip on it and we’ve got a load of the public now thinking they can make it up as they go along as well and that is anxiety provoking for people in the shielding group.” (Shield 3)

33% survey respondents strongly agreed (14%) or tended to agree (19%) that they had enjoyed spending more time at home while shielding. The availability of private gardens and outside spaces was mentioned by interviewees as a key resource for exercise and leisure throughout the shielding period; nevertheless some individuals still reported feeling trapped due to impact of shielding on their sense of freedom (Quote 2). An added complication for some interviewed parents was ensuring their children received sufficient exercise, while also personally maintaining a shielding status (Quote 3).

#### Impact on mental health

180 (90%) respondents to the survey described themselves as “somewhat” (63), “very” (51) or “extremely” (66) worried about coronavirus. Older respondents tended to be less worried about coronavirus compared with younger respondents (86% of respondents over 69 years compared with 96% of those 69 years or under were at least somewhat worried about coronavirus; X^2^ = 4.68, df = 1, *P* = 0.030).

More survey responders thought that being asked to shield had made their mental health worse (14% strongly agreed and 28% tended to agree) than thought that shielding was making their mental health better (2% strongly agreed and 4% tended to agree). When explored during interviews, various participants reported heightened emotions, worry and depression, and linked these emotions to feelings of being “locked up” for a prolonged period of time and anxiety about the future as CEV individuals (Quotes 4 to 7).

#### Depression and anxiety

**Fig. 1:**
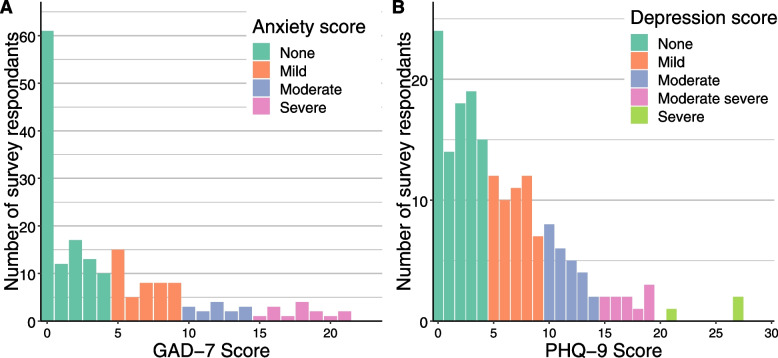
Survey results for the (A) GAD-7 and (B) PHQ-9 questionnaires (Categories indicated in the key are as follows. For the GAD-7 cut-off values used: None = 0–4; Mild = 5–9; Moderate = 10–14; Severe = 15–21. For the PHQ-9 cut-off values used were: None = 0–4; Mild = 5–9; Moderate = 10–14; Moderate severe = 15–19; Severe = 15–19. For both questionnaires a cut-off of 10 was used to indicate possible anxiety or depression, respectively.)

Twenty one percent of survey respondents had a score of 10 or above on the PHQ-9 questionnaire, indicating possible depression where treatment may be recommended (Fig. 1). 15% respondents scored 10 or above on the GAD-7 questionnaire, indicating a level of anxiety where treatment may be recommended (Fig. 1). 76% of people with a PHQ-7 score of 10 or above reported in the survey that their mental health had become worse compared to 33% with a score below 10 (X^2^ = 21.314, df = 1, *P* < 0.00001); and 72% of people with a GAD-7 of 10 or above reported in the survey that their mental health had become worse compared to 38% with a score below 10 (X^2^ = 9.863, df = 1, *P* = 0.0017). In addition, there was some correlation between people with at GAD-7 score of 10 or above and those with a PHQ-7 score of 10 or above: 73% of people with GAD-7 > 10 had a PHQ-7 > 10, and 44% of people with PHQ > 10 had a GAD > 10. 4% of survey respondents had a score of four or more on the PTSD-4 scale, suggesting they could have high likelihood of developing PTSD as a result of their experience of shielding. 28% of survey respondents had felt somewhat (19%), moderately (8%) or very (2%) angry about being told to shield, although these feelings were not discussed by interview participants.

## Discussion

Early in the pandemic the importance of providing clear, tailored advice for patients who were required to shield, alongside appropriate support, was identified [30–32]. Findings from this study showed that official shielding advice offered to CEV individuals during the first lockdown in England was deemed to be sufficient by 80% of survey responders, although interviewees criticised the delayed timing of this advice and frequently sought supplementary information to inform shielding behaviours. The individual focus of shielding advice was considered impractical and restrictive by some participants, with 66% of survey responders considering it necessary to shield with all household members. Interview participants described a spectrum of rational adaptations to the advice, with adjustments based on living situation and personal perceptions of risk [33]. Such findings suggest that it would be beneficial to engage CEV groups in the policy-making decision process for future public health emergencies; co-creation of targeted communication strategies has been shown to result in higher levels of adherence to behaviour change messages [34–36].

Organisational support (e.g., NHS, council, government, charity) was requested and received by 92.1% (35/38) of survey responders, although the type and amount of support varied between individuals, and was frequently supplemented with help from family, friends, or neighbours. These findings suggest that formal and informal support mechanisms and their coordination may benefit from being strengthened in future, potentially through involving pre-existing community-based organisations, [37] charities, volunteer organisations, and / or faith-based institutions [38]. Lessons from previous pandemics have similarly echoed the need to establish alternative means for the public to “connect” during public health emergencies, [35] with suggestions that community support could provide mechanisms for disseminating updated advice and maintaining contact with high-risk and vulnerable populations [16]. Accessing clear and accurate public health guidance via such support networks has been suggested as a protective measure against isolation and emotional distress by promoting feelings of social connection [39], which may help reduce symptoms of anxiety or depression [40–42]. Given the breadth of the CEV definition identified during the COVID-19 pandemic, research would be warranted to explore the most practical ways of utilising and aligning existing support networks for future public health emergencies.

Being formally identified as “shielding” was considered socially advantageous by some interviewees as it legitimised their socially avoidant behaviours. But for others this approach resulted in feeling “othered” as a CEV individual, [43] an issue that was further exacerbated by televised government briefings that lacked CEV specific advice, while providing reassurance to the general public that COVID-19 was most severe in those with underlying health conditions [44, 45]. Social isolation caused by the response to the COVID-19 pandemic has been shown to have a negative influence on mental health parameters [42, 46]. Of the surveyed CEV individuals, 90% were worried about COVID, with 35% agreeing that shielding was making their physical health worse and 43% reporting a negative impact on their mental health, which may have compounded these feelings of social isolation. Within this study we did not have information about participants’ mental health prior to the study, and simply being CEV may in some situations have mental health comorbidity [47]. This emphasises the importance of using communication approaches in the future that avoid implied or unintentional stigmatising of any ‘vulnerable’ group, instead providing messages framed for target groups, that are identity affirming, promote social unity, raise awareness, increase societal preparedness [10], and are delivered by the right people [48]. It is also important to note that 11% of survey respondents reported that shielding had a negative impacted on their physical health, which may be important when considering long-term motivations for self-isolation in this clinically vulnerable group.

A strength of this study was the integration of quantitative and qualitative data to triangulate information from patients identified as CEV, a methodological approach widely used for increasing the validity, breadth, and depth of mixed-method studies [49, 50]. However, our study population was 94% identified as White British, 64% were retired and 54% were aged over 69 years, therefore it is possible that our findings may not be generalisable to wider CEV populations due to demographic and experience bias. Nevertheless, our findings provide an important and valid insight into the acceptability of the shielding advice; attitudes and behaviours in response to this advice; and the impact of shielding on health and wellbeing for CEV individuals in Southwest England. Our study may also have been influenced by response bias, especially for interviewees who may have been more likely to want to discuss their shielding experiences. Last, as a cross-sectional study, pre-pandemic baseline values were unavailable, and therefore causality cannot be inferred. However, these results do demonstrate that this population did have mental health and support needs whilst shielding, which should be factored into future public health responses. Additionally, it was not possible to determine when participants had received the notification to shield, or indeed derive the reasons why participants in the study had been designated as CEV, which would impact understanding on their relative levels of risk. Centralised patient information systems with pre-emptive clinical designations would facilitate this disaggregation in future studies and contexts where tailored patient advice on a large scale is required.

## Conclusion

Individuals who are CEV are a clinically and socially heterogenous group, and the official shielding advice provided during first UK lockdown encompassed a unique combination of NPIs that were distinct from the social distancing and quarantine requirements imposed on the UK general population during the same time period. Given the uniqueness of this population and the lack of evidence on the responses of ‘shielders’, these findings provide valuable information for policy makers and healthcare professionals regarding the impacts of shielding on the wellbeing of CEV individuals during the COVID-19 pandemic, which may assist them in making preparation for future infectious disease outbreaks or other public health emergencies. Our findings emphasise the need for future public health messaging targeted at such high-risk and vulnerable populations to be co-created with relevant members of the public, tailored to health conditions, and delivered in a more targeted way with integrated assistance from existing community support systems. Furthermore, additional work should focus on factors influencing adherence to shielding advice and long-term social distancing adaptations, as well as the long-term implications of shielding for mental health and feelings of social identity.

## Supplementary Information

**Additional file 1.**

**Additional file 2.**

## Data Availability

The datasets used and / or analysed during the current study are available from the corresponding author on reasonable request.

## References

[1] National Audit Office, (2021). Protecting and supporting the clinically extremely vulnerable during lockdown. Great Britain: Ministry of Housing, Communities & Local Government, Department of Health & Social Care

[2] Department of Health and Social Care, (2020). Clinically extremely vulnerable receive updated advice tailored to local COVID alert levels. Available from: https://www.gov.uk/government/news/clinically-extremelyvulnerable-receive-updated-advice-tailored-to-local-covid-alert-levels. Accessed Sept 2022.

[3] NHS Digital, (2022). Shielded Patient List. Available from: https://digital.nhs.uk/coronavirus/shielded-patient-list#what-was-the-shielded-patientlist-. Accessed Sept 2022.

[4] Goverment Digital Services, (2021). Guidance: Get support if you’re clinically extremely vulnerable to coronavirus. https://www.gov.uk/. Accessed Dec 2021.

[5] Eraso Y, Hills S. Self-Isolation and Quarantine during the UK's First Wave of COVID-19. A Mixed-Methods Study of Non-Adherence. Int J Environ Res Public Health 2021;18(13). 10.3390/ijerph18137015 [published Online First: 20210630] 10.3390/ijerph18137015 PMC8297259 34209105

[6] Centers for Disease Control and Prevention, (2020). Nonpharmaceutical Interventions (NPIs). Available from: https://www.cdc.gov/nonpharmaceutical-interventions/index.html. Accessed Sept 2022.

[7] European Centre for Disease Prevention and Control, (2021). Non-pharmaceutical interventions against COVID-19. Available from: https://www.ecdc.europa.eu/en/covid-19/prevention-and-control/non-pharmaceutical-interventions. Accessed Sept 2022.

[8] Chen YC, Chang SC, Tsai KS (2008). Certainties and uncertainties facing emerging respiratory infectious diseases: lessons from SARS. J Formos Med Assoc.

[9] Balinska M, Rizzo C. Behavioural responses to influenza pandemics: what do we know? PLoS Curr. 2009;1:Rrn1037. 10.1371/currents.rrn1037 [published Online First: 20090909] 10.1371/currents.RRN1037 PMC2762764 20025201

[10] Bell D, Nicoll A, Fukuda K (2006). Non-pharmaceutical interventions for pandemic influenza, national and community measures. Emerg Infect Dis.

[11] Teasdale E, Santer M, Geraghty AWA (2014). Public perceptions of non-pharmaceutical interventions for reducing transmission of respiratory infection: systematic review and synthesis of qualitative studies. BMC Public Health.

[12] Margraf J, Brailovskaia J, Schneider S (2021). Adherence to behavioral Covid-19 mitigation measures strongly predicts mortality. PLoS ONE.

[13] Cava MA, Fay KE, Beanlands HJ (2005). The experience of quarantine for individuals affected by SARS in Toronto. Public Health Nurs.

[14] Hawryluck L, Gold WL, Robinson S (2004). SARS control and psychological effects of quarantine, Toronto. Canada Emerg Infect Dis.

[15] Mak IW, Chu CM, Pan PC (2009). Long-term psychiatric morbidities among SARS survivors. Gen Hosp Psychiatry.

[16] Pfefferbaum B, Schonfeld D, Flynn BW (2012). The H1N1 crisis: a case study of the integration of mental and behavioral health in public health crises. Disaster Med Public Health Prep.

[17] Carmassi C, Foghi C, Dell'Oste V (2020). PTSD symptoms in healthcare workers facing the three coronavirus outbreaks: What can we expect after the COVID-19 pandemic. Psychiatry Res.

[18] Cao W, Fang Z, Hou G (2020). The psychological impact of the COVID-19 epidemic on college students in China. Psychiatry Res.

[19] Zhang SX, Wang Y, Rauch A (2020). Unprecedented disruption of lives and work: Health, distress and life satisfaction of working adults in China one month into the COVID-19 outbreak. Psychiatry Res.

[20] Ministry of Housing Communities & Local Government, (2019). English indices of deprivation 2019 https://www.gov.uk/government/statistics/english-indices-of-deprivation-2019 [accessed Dec 2021].

[21] Kroenke K, Spitzer RL, Williams JB (2010). The Patient Health Questionnaire Somatic, Anxiety, and Depressive Symptom Scales: a systematic review. Gen Hosp Psychiatry.

[22] Spitzer RL, Kroenke K, Williams JB (2006). A brief measure for assessing generalized anxiety disorder: the GAD-7. Arch Intern Med.

[23] Ashbaugh AR, Houle-Johnson S, Herbert C (2016). Psychometric Validation of the English and French Versions of the Posttraumatic Stress Disorder Checklist for DSM-5 (PCL-5). PLoS ONE.

[24] R Core Team, (2020). A language and environment for statistical computing. R Foundation for Statistical Computing, Vienna. [Available from: URL https://www.R-project.org/].

[25] Guest G, Namey EE, Mitchell ML. Collecting Qualitative Data: A Field Manual for Applied Research. 55 City Road 55 City Road, London: SAGE Publications, Ltd, 2013.

[26] Glaser BG, Strauss AL. The discovery of grounded theory: Strategies for qualitative research: Routledge 2017.

[27] Braun V, Clarke V (2006). Using thematic analysis in psychology. Qual Res Psychol.

[28] Glaser BG (1965). The Constant Comparative Method of Qualitative Analysis. Soc Probl.

[29] Charmaz K (2006). Constructing grounded theory.

[30] Michie S, West R, Rogers MB (2020). Reducing SARS-CoV-2 transmission in the UK: A behavioural science approach to identifying options for increasing adherence to social distancing and shielding vulnerable people. Br J Health Psychol.

[31] Smith GD, Spiegelhalter D (2020). Shielding from covid-19 should be stratified by risk. BMJ.

[32] Torjesen I (2020). Covid-19: Charities call for clear advice after “utter mess” of shielding texts. BMJ.

[33] Morton K, Towler L, Groot J (2021). Infection control in the home: a qualitative study exploring perceptions and experiences of adhering to protective behaviours in the home during the COVID-19 pandemic. BMJ Open.

[34] Ghio D, Lawes-Wickwar S, Tang MY (2021). What influences people’s responses to public health messages for managing risks and preventing infectious diseases? A rapid systematic review of the evidence and recommendations. BMJ Open.

[35] Braunack-Mayer AJ, Street JM, Rogers WA (2010). Including the public in pandemic planning: a deliberative approach. BMC Public Health.

[36] Kemper S, Bongers MEJ, Slok ENE (2021). Patient and public engagement in decision-making regarding infectious disease outbreak management: an integrative review. BMJ Glob Health.

[37] Abbas MZ (2021). Public Understanding and Voluntary Responsibility to Mitigate COVID-19 Pandemic: Role of Local Community Organizations in Information Sharing and Health Literacy. Asia Pac J Public Health.

[38] Barmania S, Reiss MJ (2021). Health promotion perspectives on the COVID-19 pandemic: The importance of religion. Glob Health Promot.

[39] McKinlay AR, Fancourt D, Burton A (2021). A qualitative study about the mental health and wellbeing of older adults in the UK during the COVID-19 pandemic. BMC Geriatr.

[40] Santini ZI, Jose PE, York Cornwell E (2020). Social disconnectedness, perceived isolation, and symptoms of depression and anxiety among older Americans (NSHAP): a longitudinal mediation analysis. Lancet Public Health.

[41] Yang XY, Peng S, Yang T (2021). Changing trends of mental and behavioral responses and associations during the COVID-19 epidemic in China: a panel study. Health Educ Res.

[42] Zavlis O, Butter S, Bennett K, et al. How does the COVID-19 pandemic impact on population mental health? A network analysis of COVID influences on depression, anxiety and traumatic stress in the UK population. Psychol Med 2021:1–9. 10.1017/s0033291721000635 [published Online First: 2021/03/17] 10.1017/S0033291721000635 PMC8010290 33722329

[43] Logie CH, Turan JM (2020). How Do We Balance Tensions Between COVID-19 Public Health Responses and Stigma Mitigation?. Learning from HIV Research AIDS Behav.

[44] Abrams T, Abbot D. Disability, Deadly Discourse, and Collectivity amid Coronavirus (COVID-19). Scandinavian Journal of Disability Research 2020;22(1):168–74. 10.16993/sjdr.732

[45] Chen B, McNamara DM. Disability Discrimination, Medical Rationing and COVID-19. Asian Bioeth Rev 2020:1–8. 10.1007/s41649-020-00147-x [published Online First: 2020/09/10] 10.1007/s41649-020-00147-x PMC7471485 32901207

[46] Shevlin M, McBride O, Murphy J (2020). Anxiety, depression, traumatic stress and COVID-19-related anxiety in the UK general population during the COVID-19 pandemic. BJPsych Open.

[47] Kaplan RM, Glassman JR, Milstein A (2019). Effects of Mental Health on the Costs of Care for Chronic Illnesses. Psychiatr Serv.

[48] Neville FG, Templeton A, Smith JR (2021). Social norms, social identities and the COVID-19 pandemic: Theory and recommendations. Soc Pers Psychol Compass.

[49] Moran-Ellis J, Alexander VD, Cronin A (2006). Triangulation and integration: processes, claims and implications. Qual Res.

[50] Heath L, Wright JD (2015). Triangulation: Methodology. International Encyclopedia of the Social & Behavioral Sciences.

